# ADSC-Exosomes Alleviate MTX-induced Rat Neuronal Damage by Activating Nrf2-ARE Pathway

**DOI:** 10.1007/s12031-022-01996-x

**Published:** 2022-03-23

**Authors:** Tingting Huang, Hongfei Tong, Haixia Zhou, Juxiang Wang, Linglong Hu, Yao Wang, Zhen Huang

**Affiliations:** The Second Affiliated Hospital of Wenzhou Medical University, Wenzhou, China

**Keywords:** Adipose-derived mesenchymal stem cells, Exosomes, Methotrexate, Neuronal damage, Nrf2-ARE

## Abstract

The aim of this study was to analyze the efficacy and underlying mechanism of adipose-derived mesenchymal stem cell exosome (ADSC-exosomes)–mediated protection on methotrexate (MTX)–induced neuronal damage. We established a H_2_O_2_­induced oxidative stress model in vitro, as well as an MTX-induced neuronal damage rat model in vivo. We analyzed the effects of ADSC-exosomes on neuronal damage and Nrf2-ARE signaling pathway in rats and related mechanisms. The morphological and functional recovery of rat hippocampal neurons by ADSC-exosomes was examined by Nissl staining and modified neurological severity score (mNSS) score. The activation of Nrf2-ARE pathway effectively inhibited H_2_O_2_-induced oxidative stress. ADSC-exosomes treatment restored the activity of hippocampal neuronal cells, reduced ROS production, and inhibited hippocampal neuronal cells apoptosis. In in vivo experiments, ADSC­exosomes ameliorates MTX-induced hippocampal neuron damage by triggering Nrf2­ARE pathway, decreasing IL-6, IFN-, and TNF-a levels and TUNEL positive cells in hippocampus, and repairing hippocampal neuronal cell damage. ADSC­exosomes ameliorated MTX-induced neuronal damage and suppressed oxidative stress induced by neuronal damage through the activation of Nrf2-ARE signaling pathway.

## Introduction

As a chemotherapy drug, methotrexate (MTX) is widely employed in the treatment of various cancers and noncancerous diseases such as acute lymphoblastic leukemia and rheumatoid arthritis (Salkade and Lim 2012; Rajitha et al. 2017; Wang et al. 2018). However, it has been reported that MTX leads to cognitive impairment in patients after cessation of treatment (Fardell et al. 2010). Yang et al. (2012) have pointed out that excessive apoptosis of hippocampal neuronal cells is a mechanism of MTX-induced cognitive impairment. After MTX treatment, patient may experience impaired long-term memory, reduced arbor of the entire hippocampus, and downregulated expression of inflammatory factors (Anderson et al. 2020). MTX is also known to promote the production of reactive oxygen species (ROS) and activate oxidative stress, leading to neuronal cell apoptosis (Herman et al. 2005). Hence, there is considerable interest to find agents that can inhibit oxidative stress, inflammation, and cell apoptosis in hippocampal neuron, thereby ameliorating MTX-induced neuronal damage.

As critical mediators of intercellular communication, exosomes are nano-sized vesicles (30–150 nm in diameter) secreted by cells with lipid bilayer membrane, carrying DNA, RNA, proteins, glycans, metabolites, and lipids. These cargoes are transferred to recipient cells by exosomes and influence cell phenotype (Mathieu et al. 2019). It has been shown that exosomes excreted by adipose-derived mesenchymal stem cells (ADSCs) can alleviate oxidative stress and inflammation and restore bioenergetics (Manole et al. 2011; Arslan et al. 2013; Alexander et al. 2015; Tkach and Thery 2016). Zhao et al. have shown that adipose-derived mesenchymal stem cell exosomes (ADSC-exosomes) can promote chondrogenesis and suppress inflammation by upregulating miR-145 and miR-221 expression (Zhao et al. 2020). Nrf2 (nuclear factor erythroid 2 related factor 2) is a transcription factor that can modulate cell defense systems by regulating the expression of various detoxicant and cytoprotective genes. There is mounting evidence that the Nrf2/ARE signaling pathway can be protective against oxidative stress and neuroinflammation in neurodegenerative diseases related to cognitive impairment (Buendia et al. 2016). For instance, NRF2/ARE pathway ameliorates cognitive deficits in Alzheimer’s disease mouse models by negatively regulating BACE1 expression (Bahn et al. 2019).

Hence, this study sought to investigate the efficacy of ADSC-exosomes on neuronal damage and its underlying mechanisms. We examined whether ADSC-exosomes might have the potential to ameliorate MTX-induced neuronal damage. Furthermore, we analyzed the possibility of NRF2/antioxidant response element (NRF2/ARE) pathway as a therapeutic target for neuronal damage.

## Materials and Methods

### Cell Culture and Treatment

Hippocampal neurons were purchased from Procell (Wuhan, China). Cells seeded in 96-well plates were cultured in DMEM containing 10% FBS and 1% penicillin and streptomycin at 37 °C with 5% CO_2_. After 7 days of culture, cells were collected for subsequent experiments. The experiment was repeated 3 times for each group.

### CCK-8

To test the effect of H_2_O_2_ on cell viability of hippocampal neurons, CCK-8 assay was performed. Cells incubated in normal medium were used as control group and cells in model groups were respectively treated with 50, 100, 200, 400, or 800 µM H_2_O_2_ for 24 h.

To investigate the role of ADSC-exosomes in cell viability from hippocampal nerve cell damage induced by H_2_O_2_, cells were split into control group (cells were incubated with DMEM/F12), H_2_O_2_ group (cells treated with 200 µM H_2_O_2_ for 24 h), H_2_O_2_ + exo 10 ug/ml group, and H_2_O_2_ + exo 20 ug/ml group (cells treated with H_2_O_2_ for 24 h followed by incubation with 10 or 20 ug/ml of ADSC-exosomes for 24 h). The treated cells were rinsed with phosphate buffered saline (PBS) before addition of 100 µl of DMEM/F12 containing CCK8 solution (10 µl) into each well for 2-h incubation at 37 °C. Assay absorbance at 450 nm was measured using a microplate reader (ThermoFisher Scientific, USA). The experiment was repeated 3 times for each group.

### Dichloro-dihydro-fluorescein Diacetate Assay

ROS production in cells was determined by dichloro-dihydro-fluorescein diacetate (DCFH-DA). Cells were incubated with 500 µl of a DCFH-DA-staining solution (Beyotime Biotechnology, China) at 37 °C for 20 min. Then, the staining solution was discarded and cells washed with 3 changes of serum-free medium. Accuri C6 flow cytometry (BD, biosciences, USA) was performed to detect the fluorescence intensity of each group. The excitation wavelength was 488 nm, and the emission wavelength was 525 nm. The experiment was repeated 3 times for each group.

### Enzyme-linked Immunosorbent Assay

Specific ELISA kits purchased from Wuhan Saipei Biotechnology Co., Ltd (China) were used to determine changes in culture levels of malondialdehyde (MDA), superoxidase dismutase (SOD), glutathione peroxidase (GSH-Px), and glutathione (GSH). The levels of culture IL-6, IFN-, and TNF- were determined by corresponding ELISA kits (Abcam, China). All operations were conducted according to manufacturers’ instructions. The experiment was repeated 3 times for each group.

### Cell Transfection

The function of Nrf2-ARE pathway on oxidative stress in H_2_O_2_-induced cells was assessed through the overexpression or depletion of Nrf2 gene expression. Before H_2_O_2_ administration, cells were transiently transfected with control plasmid (BioVector), Nrf2­expressing plasmid (Transomic, Huntsville, AL, USA), Nrf2 siRNA (OriGene Technologies, Beijing, China), or the corresponding sequence scramble siRNA. The transfection processes were performed by Lipofectamine 2000 (Invitrogen). Hippocampal neuronal cells were transfected with siRNA using Lipofectamine^®^ RNAiMAX reagent (Thermo Fisher Scientific, USA) according to the manufacturer’s protocol. After 48 h of transfection, cells were collected and cell viability assays and apoptosis assays were performed. The transfection sequences are as follows: NRF2-EocR1-F: 5′-CCGGAATTCatgatggacttggaattgcc-3′, NRF2-BamH1-R: 5′-CGCGGATCC ctagtttttctttgtatccg-3′; siRNA-sense: 5′-GGUAAGUCGAGAAGUGUUUGA-3′ and antisense: 5′-AAACACUUCUCGACUUACCCC-3′.

### Animal Models

Twenty male SD rats (8–10 weeks) weighing 200–250 g were purchased from Beijing HuaFuKang Biotechnology Co., Ltd. Rats were reared in separate cages under a 12:12-light–dark cycle and were given enough food and water. Fifteen rats were subjected to intraperitoneal injection of MTX 75 mg/kg, and the rest 5 rats were injected with the equal amount of saline. In the seventh day after MTX treatment, 5 MTX-treated rats were injected with PBS, 5 were injected with PBS containing 200 ug of ADSCs­exosomes, and 5 were injected with ADSCs resuspended in PBS (1 × 106). The injections directly into rat brain parenchyma were conducted by a fully automatic brain stereotactic system (RWD, Shenzhen, China). The optimal injection location of rat hippocampus is as follows: lateral to the midline 3 mm, posterior to the bregma 3.8 mm, ventral to the bregma 3.6 mm. Hence, the rats were divided into control group, MTX + PBS group, MTX + ADSCs group, and MTX + ADSC-exo group (5 rats per group). All animal experiments were done in these 20 rats. All animal procedures were approved by the Second Affiliated Hospital of Wenzhou Medical University and conducted according to the institutional guidelines.

### ADSCs Culture

Four SD rats were sacrificed and fixed on the anatomical plate. The inguinal adipose tissues of rats were taken and cut into 1–2 mm^3^ pieces under sterile environment after being washed with PBS. Then, 0.2% type I collagenase was utilized to digest the tissue in water bath at 37 °C for 30 min. The digestion was stopped by the addition of 10% FBS. The collected cells was incubated in L-DMEM containing 10% FBS at 37 °C in a 5% CO_2_ incubator. After 72 h of incubation, non-adherent cells were discarded and the medium was replaced once every 3 days. ADSCs reaching 80–90% confluence were treated with 0.25% trypsin and passaged. The fourth-generation adherent cells were collected and trypsinized into single cells. The morphology of ADCS cells was observed with an inverted microscope, and the expression of ADSC surface markers was detected by flow cytometry.

### Isolation and Identification of ADSC-Exosomes

ADSCs were trypsinized and inoculated with 5 × 10 5 cells in 10-cm serum-free culture dishes. After culturing to fourth-generation ADSCs, the medium was collected and centrifuged at 3000 g for 15 min to remove cells and cell debris. ADSC-exosomes were isolated by density gradient centrifugation according to previous literature (Li et al. 2018a, b). The separated exosomes were identified using transmission electron microscopy, Nanosight nanoparticle analyzer and western blot. Total RNA and protein were extracted using TRIzol-LS (Invitrogen, Carlsbad, CA, USA) and exosome protein extraction kit (Invitrogen), respectively, according to the manufacturer’s protocol. ADSCs-Exo were analyzed by western blotting using the following primary antibodies: anti-CD9 (1:1000, Abcam), anti-CD63 (1:1000, Abcam), and anti-HSP70 (1:1000, Abcam). Obtain final exosomes and store at − 80 °C for use in the following studies. The experiment was repeated 3 times for each group.

### Transmission Electron Microscope

The purified ADSC-exosomes suspension (500 ug/ml) solution was fixed with glutaraldehyde. Then, 20 ul of the fixed ADSC-exosomes were added on a copper mesh (with a diameter of 2 mm). One minute later, the liquid on the edge of the copper mesh was removed. Subsequently, 35 phosphotungstic acid solution (pH = 6.8) was added on the copper mesh for 5-min staining at room temperature. Thereafter, the copper mesh was dried by an incandescent lamp. The shape and size of the ADSC-exosomes were observed under electron microscope and photographed. The experiment was repeated 3 times for each group.

### Nanoparticle Analyzer Detects the Size and Concentration of ADSC-Exosomes

PBS was used to dilute ADSC-exosomes to reach optimum concentration (1.0 × 108–2.5 × 109/mL) for detection. The diluted ADSC-exosomes was added into the sample chamber of Nanosight sample analyzer. The Brownian motion of the particles was observed and the particle size was measured by Nanosight tracking analysis software. The experiment was repeated 3 times for each group.

### TUNEL Staining

Paraffin sections were fixed in 4% paraformaldehyde overnight. Dewax in xylene for 5–10 min, change to fresh xylene, dewax for 5–10 min, absolute ethanol for 5 min, 90% ethanol for 2 min, 70% ethanol for 2 min, and distilled water for 2 min. Add 20 μg/ml DNase-free proteinase K dropwise and incubate at 20–37 °C for 15–30 min. After washing three times with PBS, put it back in the incubator for 2–10 min, then sectioned into 3–5-µm specimens. After preparing TUNEL detection solution, wash twice with PBS. Add 50 μl of TUNEL detection solution to the sample and incubate at 37 °C for 60 min in the dark. It was then washed three more times with PBS. The slides were mounted with anti-fluorescence quenching liquid and observed under a fluorescence microscope (Olympus, Japan). The excitation wavelength range used was 450–500 nm and the emission wavelength range was 515–565 nm (green fluorescence). TTUNEL Apoptosis Detection Kit (S7165, Millipore, USA) was used for TUNEL labeling according to the manufacturer’s instructions. Nuclei were counterstained with DAPI. Images were taken by a fluorescence microscope (Olympus, Japan). The experiment was repeated 3 times for each group.

### Nissl Staining

Rat hippocampal specimens fixed with formaldehyde were embedded in paraffin and cut into 4-µm-thick sections. Then, the sections deparaffinized with xylene and rehydrated in a graded series of alcohol. Samples were mounted with neutral balsam after 5 min of treatment with Nissl staining solution (Boster Biotech, China). Finally, five areas were randomly selected was observed by an inverted microscope (Leica, Germany). Nissl staining was not required for quantification. The experiment was repeated 3 times for each group.

### Neurobehavioral Tests

Modified neurological severity score (mNSS) was performed to test motion, sensation, touch, vision, balance, abnormal behavior, and muscle mass. The score ranged from 0 to 18 (0 showed the normal neurological function, and 18 represented sever brain dysfunction). The mNSS was performed on day 0, 7, 14, 21, and 28 after ADSCs or ADSC-exosomes injection. The experiment was repeated 3 times for each group.

### Western Blot

Proteins collected from cells or exosomes were separated on an SDS-PAGE gel, which included 10 µg of protein from cells or exosomes and 30 µg of protein from rats, and subsequently transferred to PVDF membranes. Then, the membranes were incubated with primary antibodies against Nrf2 (1:1000), Lamin B1 (1:2000), heme oxygenase-1 (HO-1) (1:10,000), quinone oxidoreductase 1 (NQO1) (1:10,000), ß-Actin (1:1000), heat shock protein 70 (HSP70) (1:1000), cluster of differentiation 63 (CD630) (1:1000), CD9 (1:1000), ß-tubulin (1:5000), cleaved-caspase-3 (1:500), P53 (1:10,000), B-cell lymphoma-2 (BCL-2) (1:1000), and BCL-2-associated X (Bax) (1:1000) at 4 °C overnight. Later, the secondary antibodies against rabbit and mouse were used for 1 h of incubation at room temperature. An enhanced chemiluminescence reagent (ThermoFisher Scientific, USA) was applied to visualize the results. The density of the protein bands was analyzed using software ImageJ (NIH, Bethesda, MD, USA). Anti-Nrf2, Lamin B1, HO-1, NQO1, ß­actin, HSP70, CD63, CD9, secondary antibodies against rabbit and mouse were purchased from Abcam (UK), ß-tubulin was purchased from Santa Cruz Biotechnology, Inc. Cleaved-Caspase-3, P53, BCL-2, and Bax were purchased from Shanghai Beyotime Biotechnology Co., Ltd. The experiment was repeated 3 times for each group.

### Quantitative Real-Time PCR

TRIzol reagent (Invitrogen, USA) was applied to extract total RNA from cells, followed by reverse transcription via RT SuperMix (APExBIO, USA). Later, PCR was performed using BeyoFast™ SYBR Green One-Step RT-qPCR Kit (Beyotime, Shanghai, China). The primers were shown as follows: Nrf2 forward 5′­ATGGATTTGATTGACATACTTT -3′ and reverse 5′­ACTGAGCCTGATTAGTAGCAAT-3′; HO-1 forward 5′­CTTCTTCACCTTCCCCAACA-3′ and reverse 5′-ATTGCCTGGATGTGCTTTTC-3′; and NQO-1 forward 5′-GGGATCCACGGGGACATGAATG-3′ and reverse 5′­ATTTGAATTCGGGCGTCTGCTG-3′. The fold changes were calculated by means of relative quantification (2^−ΔΔCt^ method). The experiment was repeated 3 times for each group.

### Statistical Analysis

Differences between groups were analyzed by one-way analysis of variance (ANOVA) using GraphPad 8.0 (GraphPad Software Inc., San Diego, CA, USA). The results were expressed as the mean ± standard deviation (SD). A significant difference was considered when *P* value < 0.05.

## Results

### Activation of Nrf2-ARE Signaling Pathway Inhibits Oxidative Stress

**Fig. 1:**
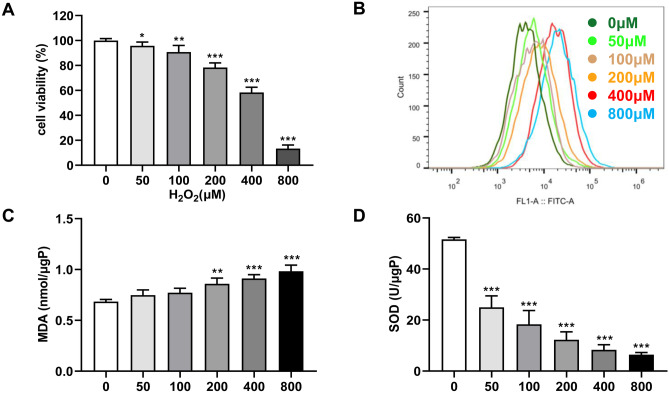
H_2_O_2_-induced oxidative stress model. **A** The viability of hippocampal neuronal cultures treated with 0, 50, 100, 200, 400, or 800 µM H2O2 was determined by CCK-8 assay, and the vertical coordinate indicated the intensity of cellular activity. **B** DCFH-DA for the detection of ROS in cells treated with different concentrations of H_2_O_2_ and the curves indicate the fluorescence intensity. **C** ELISA was performed to detect MDA content. **D** ELISA was performed to detect SOD content. The results showed that hippocampal neurons were better adapted to 200 µM H2O2. *P < 0.05, **P < 0.01, ***P < 0.001 compared with 0 µM H_2_O_2_. Number of wells/treatment; indicate data show mean ± SD. Do the same for figures below—give short summary of results. Also, indicate number of wells/treatment and what error bars designate

**Fig. 2:**
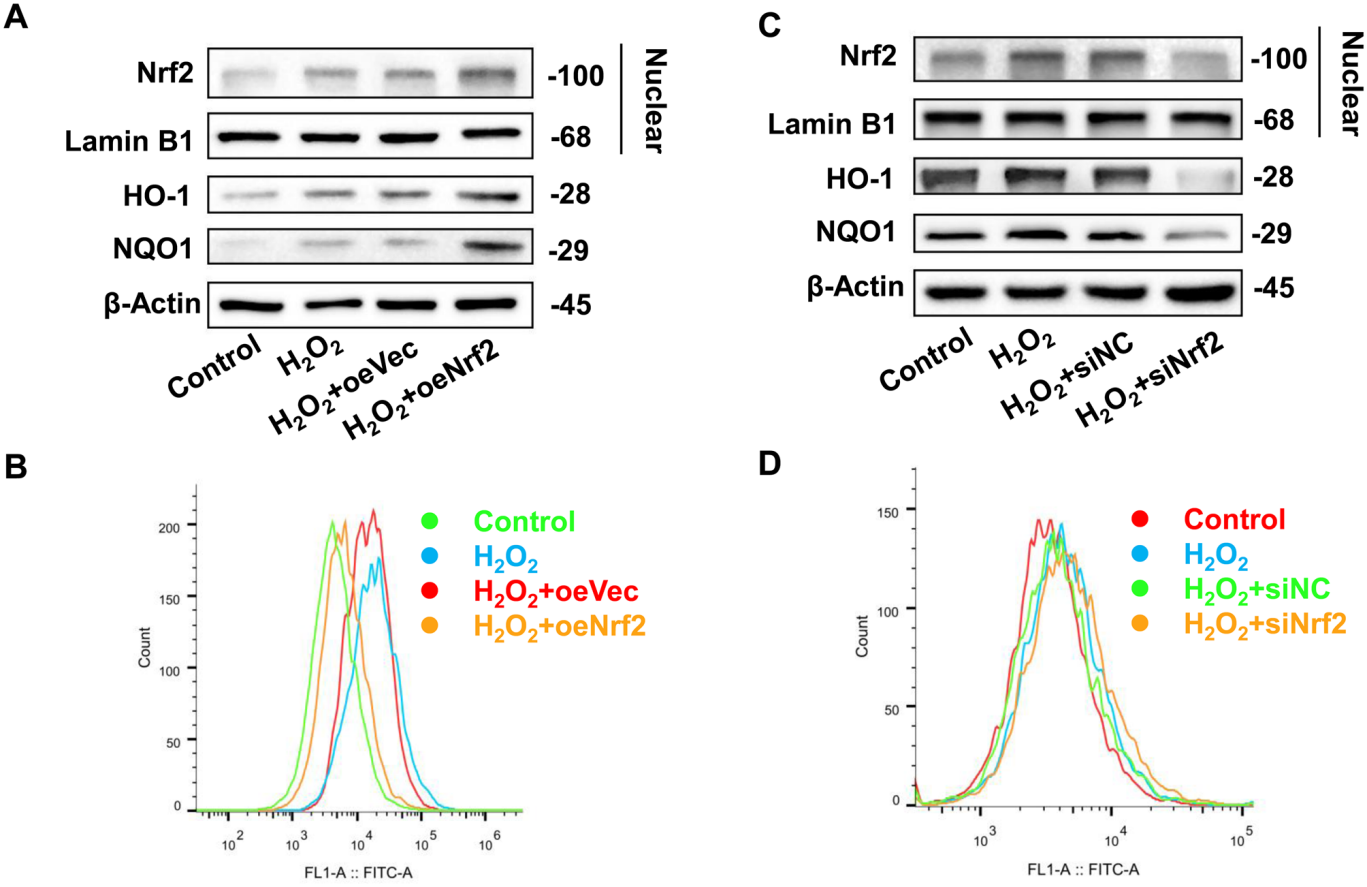
Role of Nrf2-ARE signaling pathway in H_2_O_2_-induced oxidative stress model. **A** Protein expression of Nrf2-ARE pathway after transfection with Nrf2 overexpression plasmid. **B** DCFH-DA detection of ROS expression in cells after transfection with Nrf2 overexpression plasmid. **C** Protein expression of Nrf2-ARE pathway after transfection with Nrf2 siRNA plasmid. **D** DCFH-DA detection of ROS expression in cells after transfection with Nrf2 siRNA plasmid

First, hippocampal cultures were treated with 0, 50, 100, 200, 400, or 800 µM H2O2 to assess cell viability following oxidation damage. As shown in Fig. 1A–B, 200 µM of H_2_O_2_ significantly reduced cell viability of hippocampal neurons and inhibited ROS expression. Furthermore, there was increased MDA and decreased SOD content in the cells after 200 µM H_2_O_2_ administration (Fig. 1C–D). Therefore, from these concentration studies, 200 µM H_2_O_2_ was selected for subsequent experiments. Then, we further analyzed the role of Nrf2-ARE pathway in H_2_O_2_-induced oxidative stress model. The results showed that Nrf2 overexpression significantly upregulated the protein expression of HO-1 and NQO1 and reduced ROS production (Fig. 2A–B). On the contrary, Nrf2 depletion led to reductions in HO-1 and NQO1 protein levels and an elevation in ROS production (Fig. 2C–D). Taken together, these observations suggested that oxidative stress could be suppressed by activating Nrf2-ARE pathway.

### ADSC-Exosomes Alleviate H_2_O_2_-induced Oxidative Stress in Hippocampal Neurons

**Fig. 3:**
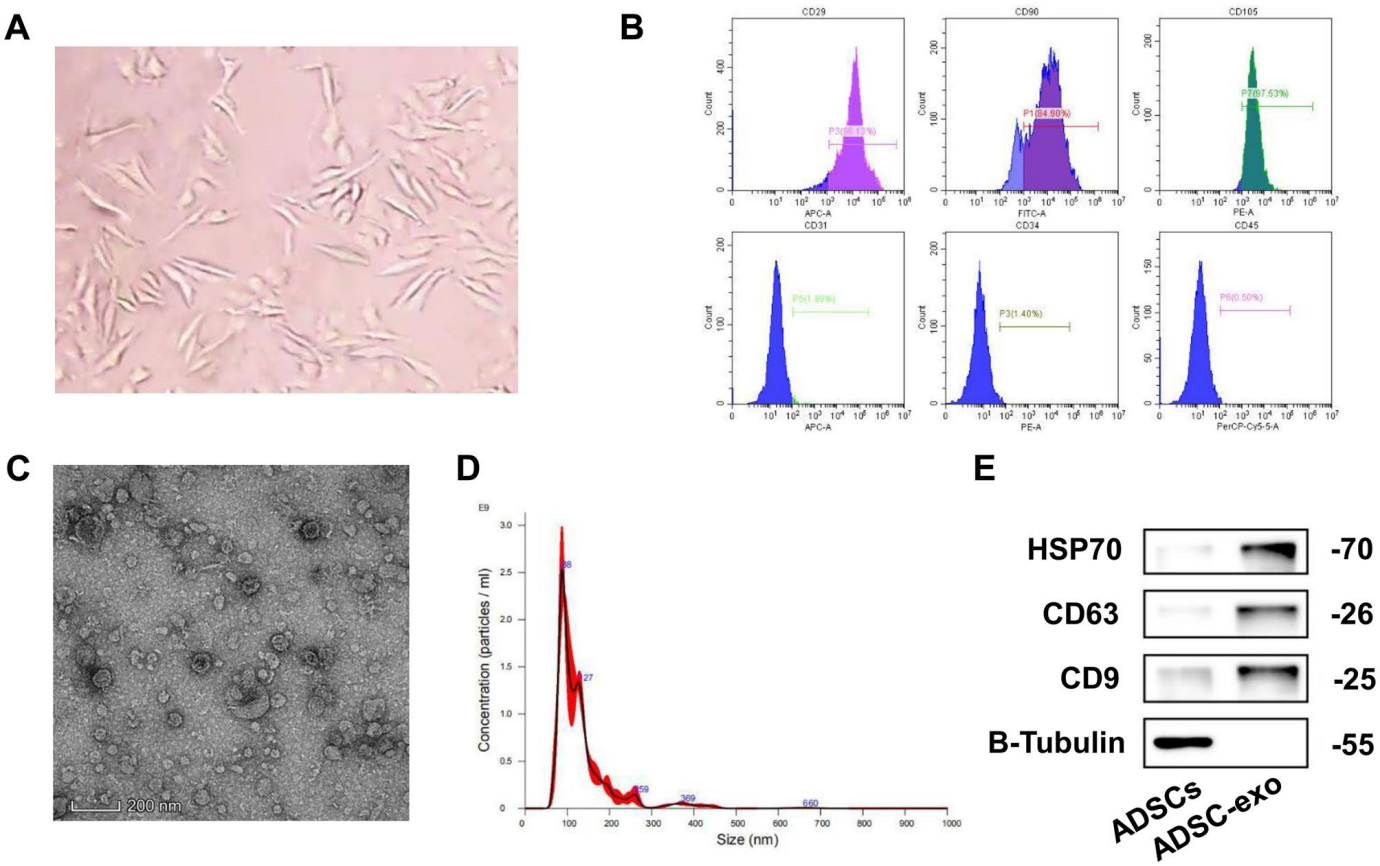
Extraction and identification of ADSC-exosomes. **A** ADSC cells were observed under microscope. **B** Cellular expression of CD29, CD90, CD105, CD31, CD34, and CD45. **C** Transmission electron micrographs. **D** Nanosight nanoparticle analysis results. **E** Western blot identification of protein expression of HSP70, CD63, and CD9 in isolated exosomes

ADSC cells are shown in Fig. 3A. The ADSC cells exhibited high expression levels of CD29, CD90, and CD105, while CD31, CD34, and CD45 levels were low (Fig. 3B). As shown in transmission electron microscope, ADSC-exosomes were round or elliptical vesicle-like shape with a complete double membrane structure and a diameter of 40–100 nm (Fig. 3C–D). In addition, Western blot results for ADSC-exosome HSP70, CD63, and CD9 confirmed that the samples collected in this study were in line with the characteristics of exosomes (Fig. 3E).

**Fig. 4:**
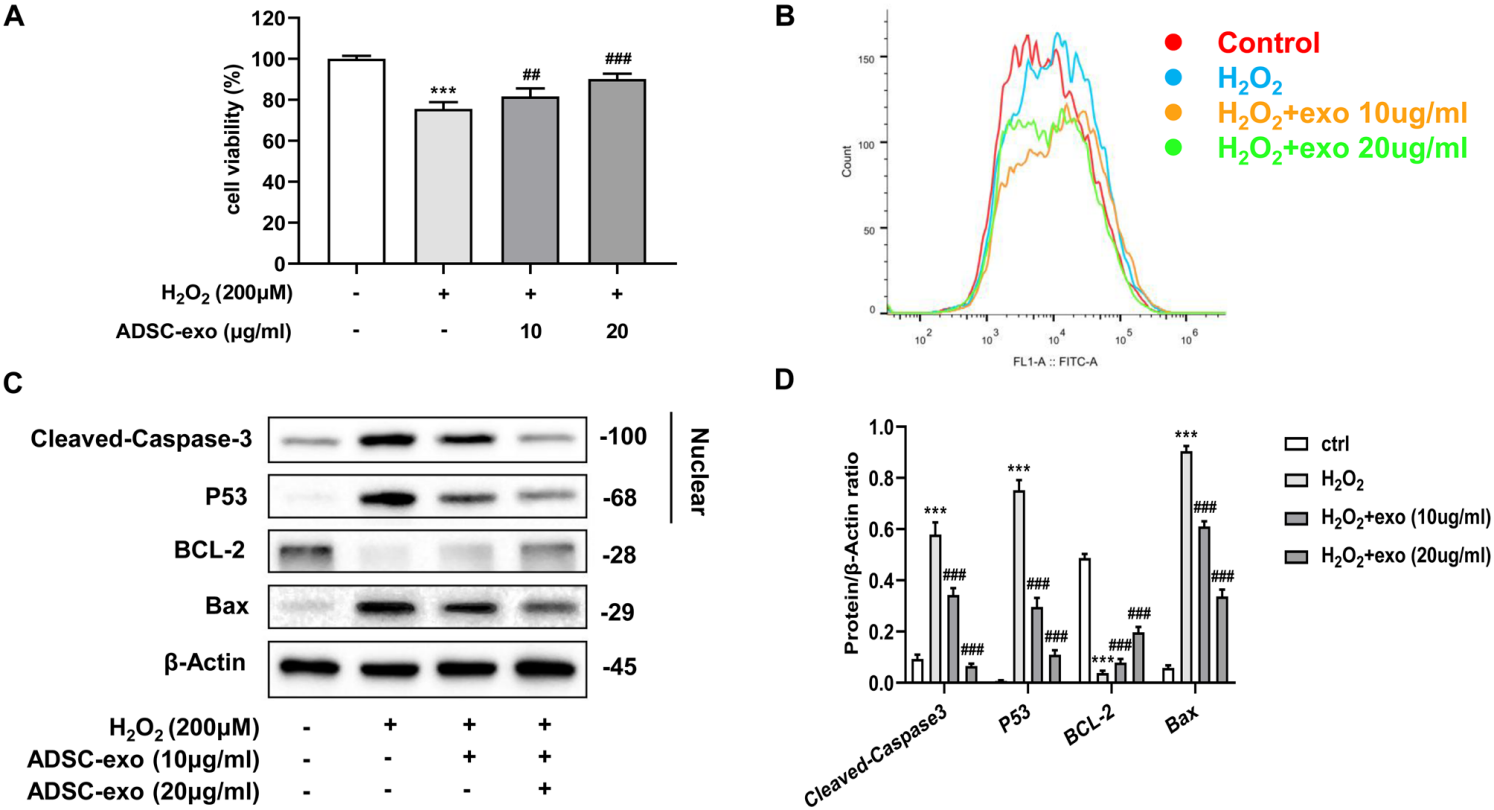
Ameliorative effect of ADSC-exosomes on oxidative stress model. **A** CCK-8 assay was utilized for determining the cell viability after ADSC-exosomes treatment. **B** DCFH-DA detection of ROS expression in each group. **C**–**D** Western blot was used to detect the protein expression of apoptotic index in each group. ^***^P < 0.001 compared with control group, ^##^*P* < 0.01, ^###^*P* < 0.001 compared with H_2_O_2_ group

The addition of ADSC-exosomes to the hippocampal cultures for 24 h, the assay revealed that ADSC-exosomes significantly reversed the cell oxidative stress damages and also reduced ROS levels induced by H_2_O_2_ (Fig. 4A–B). In concert, Western blot results showed that H_2_O_2_ significantly upregulated the expression of cleaved­caspases-3, P53 and Bax, and downregulated the expression of BCL-2; the addition of ADSC­exosomes mitigated the H_2_O_2_ effects (Fig. 4C–D). Moreover, the inhibitory effect of 20 µg/ml of ADSC-exosome was more obvious than that of 10 µg/ml of ADSC­exosome.

### The Effect of ADSC-Exosomes on Nrf2-ARE Pathwayin Vitro

**Fig. 5:**
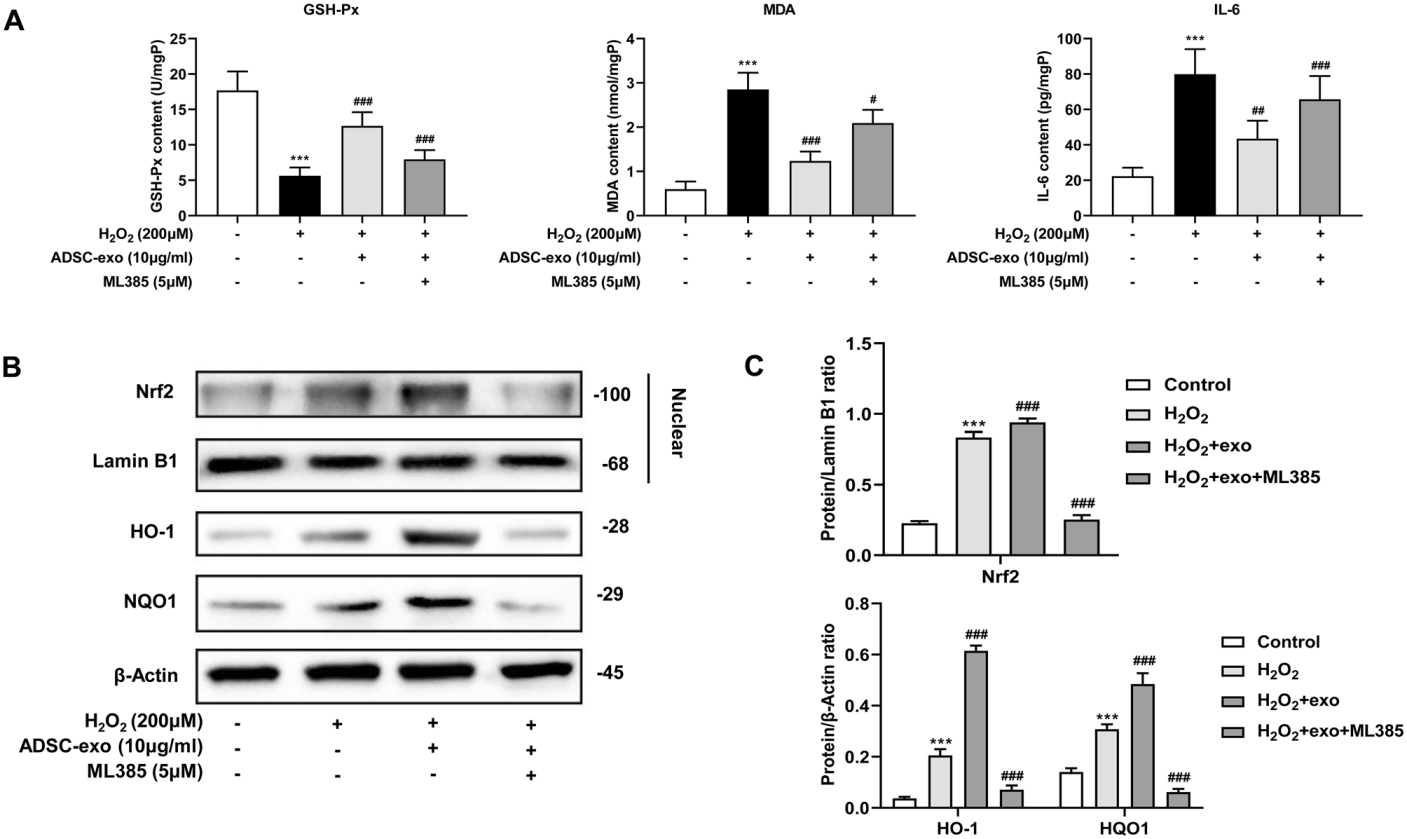
Effect of ADSC-exosomes on Nrf2-ARE signaling pathway in vitro. **A** The contents of GSH-Px, MDA, and IL-6 in cells were determined by ELISA. **B**–**C** Western blot to detect the protein expression of Nrf2, HO-1, and NQO1 in the Nrf2­ARE pathway. ^***^P < 0.001 compared with control group, ^#^*P* < 0.05, ^##^*P* < 0.01, ^###^*P* < 0.001 compared with H_2_O_2_ group

To determine the role of ADSC-exosomes on Nrf2-ARE pathway, we established four cell groups: control group, H_2_O_2_ group, H_2_O_2_ + ADSC-exo (10 µg/ml) group and H_2_O_2_ + ADSC-exo (10 µg/ml) + ML385 (a specific Nrf2 inhibitor; 5 µg/ml) group. We first measured the downstream effectors of Nrf2-ARE (GSH-Px, MDA, and IL-6) (Anuranjani and Bala 2014). The results showed that compared with the H_2_O_2_ group, the GSH-Px content in the ADSC-exosomes group was significantly elevated, but the addition of ML385 significantly inhibited this change. On the contrary, the addition of ADSC-exosomes reduced the contents of MDA and IL-6, and ML385 blocked this inhibition (Fig. 5A). To investigate whether ADSC-exosomes activate Nrf2-ARE pathway, Western blots were performed. In these studies, the protein expression of nuclear Nrf2, HO-1, and NQO1 was significantly increased after H_2_O_2_ treatment, and the addition of ADSC-exosomes further enhanced that expression. However, the protein expression levels of nuclear Nrf2, HO-1, and NQO1 in H_2_O_2_ + ADSC-exo + ML385 group were significantly lower than those in H_2_O_2_ + ADSC-exo group (Fig. 5B–C). Overall, our data showed that ADSC-exosomes could effectively enhance Nrf2-ARE pathway effects.

### ADSC-Exosomes Inhibits Oxidative Stress in MTX Rats

**Fig. 6:**
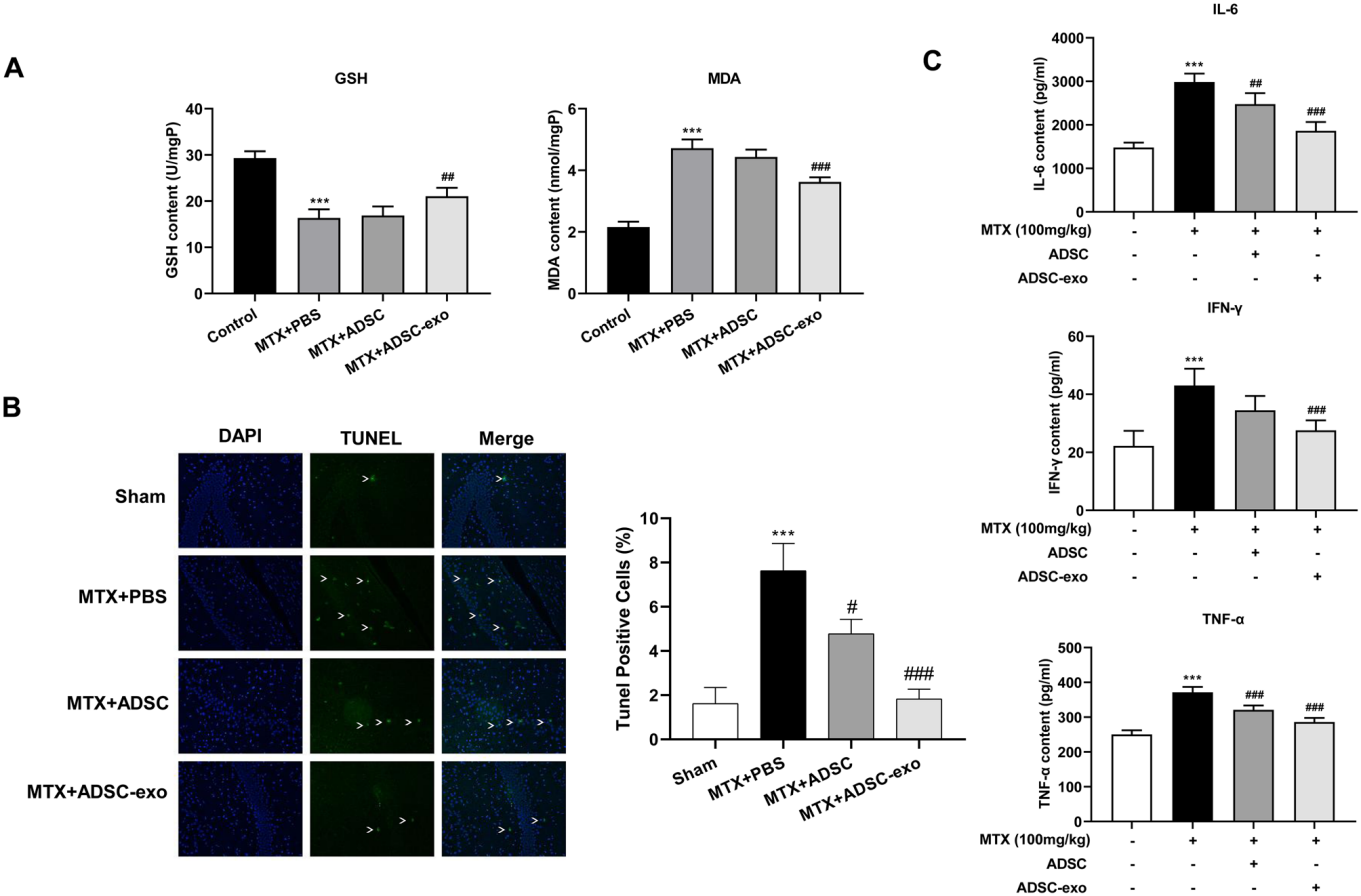
Protective effects and mechanisms of ADSC-exosomes on MTX-induced neuronal damage in rats. **A** The contents of GSH and MDA in each group were determined by ELISA. **B** Detection of hippocampal tissue using TUNEL staining and apoptosis using the TUNEL apoptosis detection kit. ^***^P < 0.001 compared with Sham group, ^#^*P* < 0.05, ^###^*P* < 0.001 compared with MTX + PBS group. **C** The levels of IL-6, IFN-, and TNF-a in each group were determined by ELISA. ^***^P < 0.001 compared with Sham group, ^##^*P* < 0.01, ^###^*P* < 0.001 compared with MTX group

The biological function of ADSC-exosomes in vivo was further confirmed using MTX-induced neuronal damage rat model. Consistent with the in vitro data, after ADSC­exosome injection (brain areas: lateral to the midline 3 mm, posterior to the bregma 3.8 mm, ventral to the bregma 3.6 mm), the GSH content in the brain tissue of rats with neuronal damage induced by MTX was significantly increased, and the content of MDA was significantly reduced (Fig. 6A). TUNEL assay revealed that MTX caused significant hippocampal neuron apoptosis. However, compared with the MTX + PBS group, the number of apoptotic cells in the MTX + ADSC and MTX + ADSC-exosome groups was significantly reduced, indicating that ADSC could inhibit MTX-induced apoptosis of hippocampal neurons, and the inhibitory effect of ADSC-exosome was better than that of ADSC (Fig. 6B). In addition, ELISA assays demonstrated elevations in the levels of IL-6, IFN-, and TNF-a in the brain tissue of MTX-induced impaired rats, which were markedly reversed after the addition of ADSC­exosomes (Fig. 6C). Collectively, ADSC-exosomes appeared to mitigate oxidative stress damages and marker expression in MTX rats.

### ADSC-Exosome Ameliorates MTX-induced Hippocampal Neuron Damage by Activating Nrf2-ARE Pathway

**Fig. 7:**
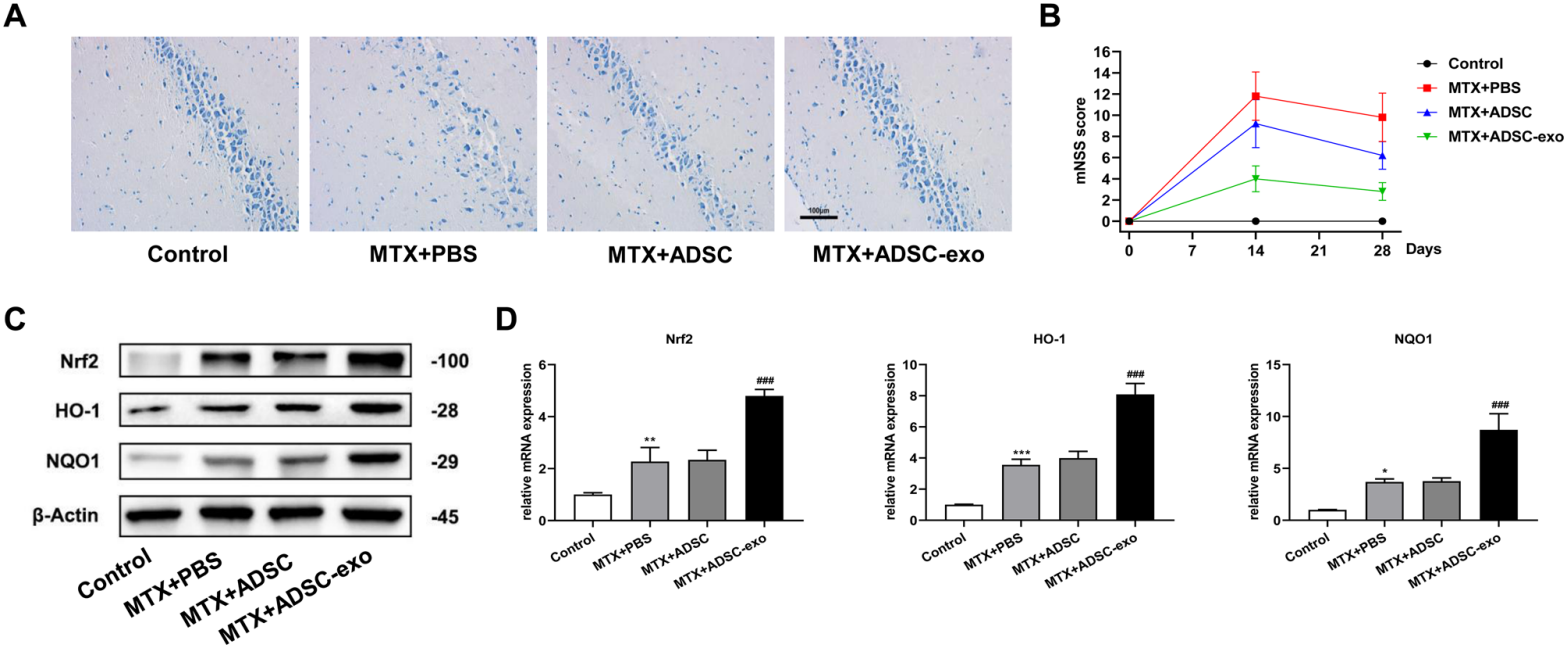
Hippocampal neurons and neurological function after MTX and ADSC-exosome treatments. **A** Morphology of rat hippocampal neurons by Nissl staining. **B** mNSS of nerve injury in rats. **C** Western blot was carried out to detect the protein expression of Nrf2, HO-1, and NQO1 in hippocampal tissue. **D** RT-qPCR detection of mRNA expression levels of Nrf2, HO-1, and NQO1 in hippocampal tissues. ^*^P < 0.05, ^**^P < 0.01, ^***^P < 0.001 compared with control group, ^###^*P* < 0.001 compared with MTX + PBS group

To determine the efficacy of ADSC-exosomes on the activity and function of rat hippocampal neurons, we evaluated the number of Nissl-stained neurons in the hippocampus. As compared with the control group, there were fewer Nissl-stained neurons in the MTX + PBS group, showing an irregular arrangement, whereas ADSCs or ADSC-exosomes treatment attenuated the MTX effects (Fig. 7A). This implied that ASDC-exosomes could rescue neuronal damage. From Fig. 7B, except for control group, mNSS scores of each group were highest by day 14; the average mNSS score of rats in the MTX + PBS group was significantly higher than that in the MTX + ADSC and MTX + ADSC-exo groups, indicating that ADSC-exosomes could alleviate nerve damage in rats. As in the in vitro studies, Western blot and RT-qPCR results showed that MTX induced the upregulated protein expression of Nrf2, HO-1, and NQO1, which was enhanced following ADSC-exosomes treatment (Fig. 7C and D). In all, our results demonstrate that ADSC-exosomes could improve MTX-induced neuronal damage by activating the Nrf2-ARE pathway.

## Discussion

MTX has been identified as an antimetabolite chemotherapeutic agent, which can produce toxic effects on the central nervous system. The resulting neuronal damages can lead to persistent behavioral and cognitive deficits. Persistent cognitive dysfunction syndrome is featured by deficits in memory, attention, multi­tasking, information processing speed, and executive function (Ellenberg et al. 2009; Koppelmans et al. 2012). Therefore, there is an urgent need to find means to mitigate MTX-induced neuronal damage. In this study, we first investigated the role of ADSCs­exosomes in MTX-induced neuronal damage. The key finding of this study was that ADSC-exosomes relieve the neuronal damages triggered by MTX through activation of the Nrf2-ARE signaling pathway.

As a primary regulator of detoxification and antioxidant systems, Nrf2 protects tissues and cells against oxidative stress by mediating ARE-regulated diverse phase II detoxification and induction of antioxidant enzymes (Cho and Kleeberger 2014; Feng et al. 2019). The expression of nuclear Nrf2 has a positive correlation with its target genes (HO-1 and NQO1) (Wang et al. 2020), which is consistent with the results we obtained in oxidative stress models. ADSCs are adipose tissue-derived MSCs with the potential of self-renewal and multidirectional differentiation and the ability to secrete hundreds of cytokines. ADSCs can improve adipose graft survival by modulating inflammatory and oxidative responses through Nrf2 and TLR4 (Chen et al. 2016). Li et al. (2018a, b) reported that ADSC-exosomes-mediated Nrf2 potentially promoted wound healing and reduced oxidative stress and apoptosis. Consistent with this, our data showed that ADSC-exosomes facilitated cell survival and inhibited the expression of oxidative stress and apoptotic markers (P53, BCL-2, and Bax) in H_2_O_2_­induced oxidative stress model. Moreover, ADSC-exosomes promoted the expression of Nrf2, HO-1, and NQO1. Overall, our results demonstrate that ADSC-exosomes can be effective for oxidative damage repair by regulating factors downstream of Nrf2-ARE pathway.

Previous studies have shown that MTX-triggered inflammation, oxidative stress, and apoptosis of neurons in the hippocampus can contribute to the development of cognitive impairments (Genestier et al. 2000; Seigers et al. 2008; Wu et al. 2017). In line with previous studies, the increased level of inflammatory factors IL-6, IFN-, and TNF­ and enhanced cell apoptosis in hippocampal tissues were observed in the MTX-induced rat model. Conversely, MTX treatment reduced GSH content and increased MDA content, which was in agreement with Al-Majed et al. (Al-Majed et al. 2004). As reported, ADSC-exosomes have therapeutic potentials in many fields, such as wound healing, neuroprotection, and protection of cells and tissue from oxidative stress (Bucan et al. 2019; Liu et al. 2019; Ma et al. 2019). Liu et al. showed that ADSC-exosomes protected cardiac myocytes from oxidative stress (Liu et al. 2019). In the present study, ADSC-exosomes promoted cell viability of hippocampal neurons after oxidative stress, inhibited apoptosis, and effectively protected cells from oxidative stress–induced damages. The same results were also found in in vivo with ADSC-exosomes in an MTX-induced rat model, suggesting that ADSC-exosomes might be a promising agent for ameliorating MTX-induced neuronal damage. Moreover, studies have shown that regulation of the Nrf2-ARE pathway can effectively improve neuronal damage caused by high salt, cholesterol, diabetes, and Alzheimer’s disease (Husain et al. 2018; Chen et al. 2019; Tian et al. 2019). Our in vivo data revealed that ADSC-exosomes activated the Nrf2-ARE pathway in hippocampal neural tissue, thereby promoting the expression of Nrf2, HO-1, and NQO1. These findings show that hippocampal neuronal number and function in MTX-impaired rats can be markedly enhanced after the addition of ADSC-exosomes.

## Conclusion

This study illustrates that ADSC-exosomes can suppress MTX-induced oxidative stress in hippocampal neurons by activating Nrf2-ARE signaling pathway. Additionally, Nrf2­ARE signaling pathway is closely implicated in the alleviation of MTX-induced neuronal damage in the hippocampus. Overall, ADSC-exosomes appear to possess therapeutic potential for MTX-induced neuronal damage.

## Data Availability

The data used to support the findings of this study are available from the corresponding author upon request.
